# Ethical issues of the use of AI-driven mobile apps for education

**DOI:** 10.3389/fpubh.2022.1118116

**Published:** 2023-01-11

**Authors:** Blanka Klimova, Marcel Pikhart, Jaroslav Kacetl

**Affiliations:** Department of Applied Linguistics, Faculty of Informatics and Management, University of Hradec Kralove, Hradec Kralove, Czechia

**Keywords:** artificial intelligence, mobile apps, ethics, ethical principles, education

## Abstract

Nowadays, artificial intelligence (AI) affects our lives every single day and brings with it both benefits and risks for all spheres of human activities, including education. Out of these risks, the most striking seems to be ethical issues of the use of AI, such as misuse of private data or surveillance of people's lives. Therefore, the aim of this systematic review is to describe the key ethical issues related to the use of AI-driven mobile apps in education, as well as to list some of the implications based on the identified studies associated with this research topic. The methodology of this review study was based on the PRISMA guidelines for systematic reviews and meta-analyses. The results indicate four key ethical principles that should be followed, out of which the principle of algorithmovigilance should be considered in order to monitor, understand and prevent the adverse effects of algorithms in the use of AI in education. Furthermore, all stakeholders should be identified, as well as their joint engagement and collaboration to guarantee the ethical use of AI in education. Thus, the contribution of this study consists in emphasizing the need for joint cooperation and research of all stakeholders when using AI-driven mobile technologies in education with special attention to the ethical issues since the present research based on the review studies is scarce and neglected in this respect.

## Introduction

At present artificial intelligence (AI) is an indispensable part of people's lives and affects all fields of human activity, including education where it helps to enhance personalized learning and thus makes learning more student-centered in the form of using exploratory learning, collaborative, automatic assessment systems (1, 2), mobile game-based learning (3, 4) or conversational chatbots for developing foreign language skills (5–7). However, there are many aspects of the use of AI that need to be researched as the lack of data in these areas is still an issue, such as the impact of AI-driven tools to enhance human cognition or second language acquisition. The facts we know are that the use of AI technology in classes not only contributes to students' learning but can also reduce in some respect a teacher's workload and can improve students' learning and their learning results (8–10). However, fortunately, AI-driven technology cannot replace teachers' pedagogical work in any case (11) since AI technology does not know which methods suit best to meet students' learning needs.

Despite the undeniable benefits that AI technology brings for both students and teachers, there are certain risks and threats associated with ethical issues and these risks should be carefully evaluated by both conceptual and empirical studies that will clearly delineate where the potential threats could be. One of these major risks is privacy or the lack of it. AI technology based on algorithmic applications intentionally collects human data from its users and they do not specifically know what kind of data and what quantities of them are collected. Although legislatively (in many countries or geographical/political regions, such as the European Union) user consent is required before using any AI technology, the user actually does not know what is happening with his/her data in the system (12). Therefore, AI technology companies should minimize this data and aim to include only the information that can enhance student learning (1).

In addition, using, for instance, chatbots for developing foreign language speaking and writing skills, indicates another problem and that is monitoring students' ideas, which might consequently decrease student engagement in using this tool since s/he does not want to be tracked or even stalked for his/her ideas [cf. (13)]. This aspect is also related to students' autonomy, i.e., the ability to govern their own learning since the use of algorithms can make predictions about their actions based on provided information input by students (14). As Reiss (15) puts it, *within every AI system there are the fruits of countless hours of human thinking*. Furthermore, another risk is connected with gender bias, for example, when using machine translation tools (16) that could actually create an environment that is not considered fair from the gender perspective.

Therefore, to reduce these risks, the European Commission (17) in October 2022 published a set of ethical guidelines for primary and secondary teachers, as well as for school leaders in order to effectively integrate AI technology and data into school education and raise awareness of their possible threats. The ethical use of AI and data in learning, teaching, and assessment is based on four key ethical considerations, which include human agency, fairness, humanity, and justified choice. In addition, the document lists new competencies of educators for the ethical use of AI technology and data for educational purposes, such as being able to critically describe the positive and negative impacts of AI and data use in education or being able to understand the basics of AI and learning analytics.

Therefore, the aim of this review is to describe the key ethical issues related to the use of AI-driven mobile apps in education and draw the attention of the academic community to these issues that might be set aside in the quest for research outcomes. The following research questions were formulated:

1. *What are the major ethical issues that could be observed when using AI-driven mobile apps for educational purposes?*
2. *What are the future lines of research related to the given topic that can be obtained from the studies available?*

## Methodology

To obtain the answer to the research question, the study strictly followed the PRISMA methodology for systematic reviews and meta-analyses. This analysis was used to delineate a major trajectory of the given topic so that further implications could be shown. The following inclusion and exclusion criteria were followed.

### Inclusion criteria

- All studies focusing on the research topic.
- Published between January 2018 and December 2022, i.e., last five years.
- Scopus and Web of Science databases.
- Peer-reviewed and only English-written journal articles were included.
- Search terms were applied in the title, abstract, or keywords of the articles.
- Open access journals.

### Exclusion criteria

- Published earlier than 1 January 2018.
- AI-driven apps that are not used for educational purposes.
- Other (less reputable) databases.
- Other languages.
- Other than open access articles (such as gold and hybrid access, etc.).

### Search string

The following search string was applied to create a dataset of all relevant articles.

(“AI” OR “artificial intelligence”) AND education AND ethic^*^.

As the search string was rather wide, it was necessary to manually eliminate all nonrelevant articles to yield only those that significantly contribute to the topic. The initial search using this search string generated 118 documents from Scopus and 467 studies from the Web of Science. After applying all inclusion and exclusion criteria and removing duplicates, 44 studies could be considered to be analyzed. The authors also conducted a backward search, i.e., they searched the references of detected studies for relevant research studies which could be missed during their research. This generated another 2 studies. Thus, altogether 46 experimental studies were identified for the full-text analysis. After this initial screening, all the studies were carefully checked for their relevance to the topic by the research team and only 8 documents remained to be included in this analysis as they all represented breakthrough ideas that pertain to the topic and bring a novel and unbiased approach.

## Results

The following studies have been yielded as they contain a clear systematic approach to the topic and they fully focus on the ethical issues of AI in mobile apps (14, 18–24). The other detected texts only included more or less superficial comments on ethics, touching only on isolated issues, such as privacy [e.g., (25–28)]. These studies are interesting and important, however, they could not be included in this specific conceptual research frame.

The chosen studies can be further subdivided into those that address purely educational issues related to AI (19, 21, 22, 24) and those that discuss the use of AI in medicine (20, 23) and could also be included in this study, while Leimanis and Palkova (18) address ethical issues of AI in medical education. The remaining study (14) deals with the ethics of AI in various disciplines, with machine ethics, a subfield of the ethics of AI, as a focal point but it provides insightful comments related to education, therefore, it also had to be included in this review. The summary of the key findings are in Table 1.

**Table 1:** An overview of the key findings from the detected studies.

**References**	**Field**	**Objective**	**Ethical principles mentioned**	**Findings**
Leimanis and Palkova (18)	AI ethical issues in healthcare education	Draw attention to ethics as an international problem and challenge global standards or ethical setting instruments	Seven key requirements that AI systems should meet in order to be deemed trustworthy: human agency and oversight, technical robustness and safety, privacy and data governance, transparency (the data, system and AI business models should be transparent), diversity, non-discrimination and fairness, societal and environmental well-being (AI systems should benefit all human beings, including future)	Numerous institutions create their own standards-the policies differ significantly in content and application; multi-stakeholder collaboration is required to optimize accountability, transparency, privacy, and impartiality to create trust.
Henry and Oliver (19)	Ethics of engaging with artificial intelligence (AI) in higher education—ethical and trustworthy AI	Show that ethics should not be understood only as abstract values or design decisions, but as socio-technical achievements—political and practical “doings,” enacted in the practices of students, teachers, and corporations.	Restorative/reparative justice values such as obedience, the rule of law, and deterrence Fairness, accountability and transparency explicability, non-harmful use, responsibility and integrity	The ethics of using AI in education are political, involving the distribution of power, privilege, and resources. Immediate need for restorative justice against the slower temporality of systemic failure. Create new relationships between universities, students, businesses, algorithms, and the idea of academic integrity.
Hu et al. (20)	(Current barriers to) AI adoption in patient care (medicine)	Warn of the risks posed by AI due to its non-transparency and inherent potential for harm when used as a decision-making tool. Prove that the role of the physician (humans) likely remains paramount to (clinical) decision-making in the near future.	Deontology, nonmaleficence, utilitarian conflict, and beneficence. Utilitarian conflict of beneficence in deciding the extent to which it is acceptable to use an AI algorithm that may be more accurate and benefit certain subgroups at the expense of others. Deontological conflict to adhere to nonmaleficence. If we know there is a high likelihood of increasing disparity despite the beneficial aspects of AI, the application of AI would be unethical.	Substantial data bias may lead to unforeseen disparities in patient care as AI may stratify based on unintentional subgroups. Interdisciplinary collaboration between data scientists, data stewards, clinicians, and healthcare workers is crucial to developing a risk liability and quality improvement system before AI can serve as a medical decision-maker. AI is capable of identifying hidden features within data that can be leveraged to improve decision-making, but it is not without potential risk and needs to be deliberated by all stakeholders
Javed et al. (21)	Education: they analyzed 166 syllabi of AI ethics courses at 105 universities around the world.	Uncovers topics in teaching ethics in AI courses and their trends related to where the courses are taught, by whom, and at what level of cognitive complexity and specificity. Analyze patterns of teaching AI ethics and critically assess their implications.	Philosophy-related syllabi often include the study of classic ethical frameworks (e.g., utilitarianism, deontology, virtue-based ethics)	Department-wise disciplinary distribution of AI ethics courses: computer science, humanities, multidisciplinary, and law. An essential solution stressed for decades by educational, governmental, and industrial organizations for addressing problematic issues has been to incorporate ethics into teaching AI to tech professionals and future AI practitioners ranging from raising ethical awareness to developing concrete skills for the implementation of ethical guidelines.
Renz and Vladova (22)	AI in Education, learning, and teaching	Introduce the concept of human-centered AI (HCAI, i.e., AI under human control), which in line with human values without risks to humanity. It uses “design-for-values” approach (aims at making /incorporating moral values part of technological design, research, and development)	Human values, human rights, human dignity, and human freedom are at the center of AI design	HCAI teaming (integrating people with AI assistants) model of education. Now is the right time to consider value-conscious design principles in developing human-centered and responsible AI that addresses social, legal, and moral values prior to and during the technology development process.
Solomonides et al. (23)	American Medical Informatics Association's (AMIA) AI life cycle principles	Define and provide a rationale for principles that should guide the commission, creation, implementation, maintenance, and retirement of AI systems as a foundation for governance throughout the life cycle.	Requirements of practice and research in medicine and healthcare: beneficence (AI is designed explicitly to be helpful to people who use it, or on whom it is used, and to reflect the ideals of compassionate, kind, and considerate human behavior), nonmaleficence (“Do No Harm,” every reasonable effort to avoid, prevent, and minimize harm or damage to any stakeholder), autonomy, and justice (equity in representation in and access to AI, data, and the benefits of AI; remedy in the event of harm resulting from the use of AI; the affirmative use of AI to support social justice) comes first. A set of principles follow from the creation and engineering of AI systems: explainability of the technology in plain terms; interpretability, that is, plausible reasoning for decisions; fairness and absence of bias; dependability, including “safe failure”; provision of an audit trail for decisions; and active management of the knowledge base to remain up to date and sensitive to any changes in the environment.	Introduce AI judiciously, in the appropriate environments, and in accordance with the principles outlined here. Principles require benevolence—aiming to do good through the use of AI; transparency, ensuring that all assumptions and potential conflicts of interest are declared; and accountability, including active oversight of AI systems and management of any risks that may arise. Stakeholders must be identified and consulted.
Shih et al. (24)	AI (including AI ethics) course for students with non-engineering backgrounds	Attempts to answer the following two questions: • Does the present situated-learning-based course have an effect on students' understanding of AI, AI teamwork, and attitudes toward AI? • Does the present course enhance students' awareness of AI ethical issues?	Ethical issues that arise in the use of AI include transparency, fairness, responsibility, and sustainability	Learning about ethical issues related to AI requires diverse perspectives from different fields of expertise. AI understanding and attitude toward AI can predict learners' awareness of AI ethical issues. The design of the course activities helped students pay more attention to the ethical issues.
Stenseke (14)	AI ethics across a diverse set of disciplines Machine ethics (ME)—a subfield of AI ethics—seeks to implement ethical considerations into AI systems	Explore the gap between ethics and technology and look for ways to reconcile the conflict between two discipline-specific approaches to machine ethics: the philosophical approach and the engineering approach Q: Whether and to what extent machines can or should be moral.	What is good; What does it mean to do good/to be good? Deontology, consequentialism (compared to utilitarianism, it does not specify the desired outcome). Determine what is moral in particular cases (applied ethics), advance general standards of what is moral (normative ethics), or explore the meaning and nature of morality (metaethics). Phenomena— e.g., moral behavior, moral cognition, moral values, or moral environments. Concepts like consciousness (phenomenal consciousness is central for the moral agency), autonomy (free will—Kantian tradition), and rationality are central to ME, Aristotle's animale rationale), “empathic rationality” capable of moral imagination and reflective equilibrium (Purves et al., 2015), Humean empiricism (“reason is the slave of passions”), and Kantian rationality (according to the law of the autonomous will) allegedly ethical machine?	Two main types of ME: (1) The philosophical approach to machine ethics (PME) weak obligation/advice—ethically justified—the conceptual exploration of what computer systems ought to do, and what systems ought to be built. (2) The engineering approach to machine ethics (EME) possibility- technically feasible—the exploration of what kind of morality can be implemented in computer systems, and what moral systems can be built. Work in machine ethics is propelled and shaped by conflicting disciplinary perspectives (philosophy and computer science) that lead to confusion on the prospect of machine morality—ethicists and engineers should strengthen and enrich their views with perspectives beyond their own discipline.

From a theoretical point of view, the key areas of ethics are at least mentioned in the texts studied. Stenseke (14) explains that ethics asks questions about what is good in particular cases, which points to *applied ethics*, then proposes general norms, which is what *normative ethics* is for, and explores the nature of morality, which is the topic of *metaethics*. The most widespread ethical theories are mentioned in at least one of the examined texts. These include *deontology* (14, 20, 21), *utilitarianism* [(14, 20, 21), *consequentialism* (14), *virtue ethics* (14, 21)]. These conceptual clarifications seem relevant and provide the reader with a necessary theoretical background that can be further extended into a practical application of this theoretical framing.

In terms of ethical principles that most often relate to the ethics of AI, the analyzed texts [e.g., Solomonides et al. (23) list the four prima facie ethical principles of beneficence, nonmaleficence, autonomy, and justice] include these that revolve around the following ones:

- *Beneficence* (beneficence, benevolence, nonmaleficence, do good, be good, goodness).
- *Accountability* (accountability, (risk) liability, responsibility, but also trust, explainability, interpretability, auditability, trustworthiness, transparency, sustainability, dependability).
- *Justice* (equality, justice, fairness, equity, no bias, no discrimination).
- *Human values* (human rights, dignity, freedom, autonomy, moral behavior, consciousness, rationality).

The texts studied bring some remarkable findings that seem to be very important to the understanding of the current situation. First, the most significant finding, included in six studies (14, 18–20, 23, 24), is that interdisciplinary, multi-stakeholder collaboration that takes into account different perspectives across disciplines is essential for establishing globally acceptable standards of AI ethics. These stakeholders include end-users and developers of AI systems, as well as other organizational and societal stakeholders, including manufacturers and indeed anyone who comes into contact with AI systems (23). Currently, there appear to be various barriers to such collaboration, as policies vary in content and application from institution to institution [e.g., Leimanis and Palkova (18)]. Perspectives also differ significantly from discipline to discipline (e.g., social science, philosophy, engineering, law) as ethicists and engineers rarely find common ground (14).

Javed et al. (21) studied the disciplinary distribution of AI ethics course delivery by the department and argue that AI courses are mainly delivered by computer science and humanities departments, but also by law departments, and some courses are multidisciplinary, i.e., offered by at least two different departments. The same authors recognize three basic approaches to teaching AI ethics, namely Build (engineering and technical aspects—trustworthy technical solutions), Assess (multidisciplinary teams focused on philosophy and application—judgments based on fundamental principles), and Govern (humanities and law disciplines focused on developing general knowledge—stakeholder protection). They add that holistic teaching that removes disciplinary, topic-oriented, and other barriers should be pursued (21). In the same vein, Stenseke (14) emphasizes the importance of avoiding a narrow disciplinary perspective by analytically understanding the different ways in which each discipline understands underlying concepts, conducts its research, and produces results.

Second, based on the texts studied, it can be assumed that the most promising areas for the development of AI ethics are *medicine, education, and engineering* (i.e., engineering directly related to AI). However, we cannot see these fields as necessarily strictly separated, but rather as overlapping or intertwined. This is especially so because interdisciplinary collaboration is seen as highly beneficial in the field of AI ethics as it is repeatedly supported by various research findings [see e.g., (14, 18–24)]. Engineering primarily encompasses the field of AI system manufacturing and includes all those involved in the entire AI system life cycle. It mainly covers the technical aspects related to the existence and use of AI. Medical ethics, because of its systematic nature and thoroughness based on a long tradition, carries the qualities necessary for the development of ethical theory and practice in the field of AI applied, among other areas, to education. Education actually brings all stakeholders together, because everyone can be educated in the ethics of AI. Education opens up new horizons for students, encourages them to ask new questions, and is aware of the different short-, medium- and long-term possibilities related to AI.

Third, there is a fundamental potential conflict within the ethics of AI. It consists in answering the question of whether to prioritize human-centered AI (HCAI, AI under human control) (22) or machine ethics (ME) (14). The former puts humans in the position of decision-makers, while the latter sees the future in (potential) AI being ethical in its own right without (unnecessary) human intervention. Stenseke (14) considers machine ethics as a subfield of AI ethics and adds that there are two types of machine ethics, namely the philosophical approach to machine ethics (PME) and the engineering approach to machine ethics (EME). While the former is based on weak *obligations or advice*—what AI *ought to* do and what AI systems *ought to* be created, the latter focuses on *possibilities or technical feasibility*—what morality *can* be implemented in AI and what moral AI systems *can* be created.

At least for now, we believe that AI should be controlled by humans. Although AI systems are (usually) designed for beneficial purposes, they can go awry and behave in ways that are unexpected, unclear, and counterintuitive from a human perspective (23). Nevertheless, theoretical discussions and research regarding artificial moral agents (AMAs, i.e., autonomous machines capable of making human-like moral decisions) are already underway (14).

Fourth, Solomonides et al. (23) and to some extent Renz and Vladova (22) consider the entire life cycle of AI systems. The main idea is that ethical considerations should provide ethical principles and guidelines for all activities related to the entire life cycle of AI systems, from the specification or commissioning to the creation and design, implementation, and maintenance, to the decommissioning of AI systems. Several key recommendations stem from this. One is the continuing engagement of identified stakeholders (23). Another is the application of an AI-specific ethical principle—algorithmovigilance, which is essentially the ongoing oversight of AI systems (23). Last but not least, social, legal, and moral values need to be consciously taken into account at all times (22).

## Discussion

AI-related ethics, as a practical human endeavor that is studied with the help of theoretical insight, can provide very insightful comments and ideas that need further verification from a practical perspective. Moreover, it requires multi-stakeholder, interdisciplinary collaboration that embraces different perspectives because this is the only possible way how to obtain reliable results that could be further utilized in education, medicine, and other fields that utilize AI and other digital tools that can potentially pose a threat to the human mind and therefore they must be studied from various perspectives, one of them being ethics.

Fortunately, many authors (14, 18–20, 23, 24) are already aware of the need to respect and employ diverse perspectives on AI ethics when designing, programming and creating mobile apps for various purposes, including education. In the case of education, one must not forget that these tools will be widely used by children and the younger generation as they are in the process of formal and informal education and they will thus be massively impacted by these technologies. Despite the fact that they belong to the technologically savvy Gen Z and Millennials (29), they are still, or even more, vulnerable to the threats they are exposed to, such as surveillance or sexual harassment. It also seems that ethical-related issues will gain in their momentum when various kinds of virtual, augmented, and mixed reality will become an everyday part of our lives. For all these reasons, interdisciplinary interconnectedness seems crucial as it will be necessary to connect technological aspects with ethical considerations, which will enable our survival as a society and also the individual members of it (30).

Furthermore, when thinking about interdisciplinarity, Stenseke (14) suggest ways to make interdisciplinary integration and collaboration more effective by exploiting the possibilities of different perspectives while being aware of their limitations. Indeed, the perspectives differ based on disciplines, e.g., social science, philosophy, engineering, and law, but also based on time, i.e., short-term considerations as well as potential long-term risks (14). While all stakeholders can indicate various practical ethical problems related to AI and offer possible solutions, ethicists are there to refine all of this with respect to ethical theory and to point out possible pitfalls of a lay perspective. Still, even if there are (globally accepted) ethical guidelines for AI [e.g., Javed et al. (21) mention the ACM ethics guidelines—see https://ethics.acm.org/code-of-ethics/software-engineering-code/], they will not necessarily lead to the ethical functioning of AI. Businesses and institutions can abuse or misuse them as ethics-washing, i.e., a strategy to cover up unethical behavior (14).

The ethical functioning of AI also includes issues of AMAs and machine ethics. The question of whether and to what extent machines should/could be held accountable is extremely difficult to answer. We are leaning more toward human-controlled artificial intelligence, but technology is evolving so rapidly that it is almost impossible to predict what machines or artificial intelligence will be able to do in a few decades. Despite this, as long as stakeholders raise and discuss potential problems, AI ethics should be able to come up with relevant solutions to the looming problems posed by human interaction with AI (31).

The major limitation of this study seems to be a lack of clear-cut empirical research into the topic of AI and its ethical issues in relation to education. There is a need for at least descriptive studies that could analyze the current situational issues related to ethical issues in mobile apps as they appear based on the authors' everyday observations when using them. The volume of data available is surprisingly extremely limited to draw any concise conclusion and many authors come to very daring and unjustified suggestions regarding the implementation of AI in all teaching practices without realizing its potential problems and dangers [such as (32)]. However, these conclusions, at least preliminary, are needed and they should be considered a must for further development of this vast area of AI in mobile apps for educational purposes. If we ignore the topic's urgency, we could easily put the whole generation of young users of these apps in danger and the risks related to them are unrepairable.

In conclusion, the research questions set at the beginning provide the following summary: *The major ethical issues that could be observed when using AI-driven mobile apps for educational purposes* include key four ethical principles that should be followed—beneficence, nonmaleficence, autonomy, and justice. In particular, the principle of algorithmovigilance should be considered in order to monitor, understand and prevent the adverse effects of algorithms in the use of AI in education [cf. (33)]. Furthermore, all stakeholders should be identified, as well as their joint engagement and collaboration to guarantee the ethical use of AI in education.

As far as the second research question is concerned, i.e., *the future lines of research related to the given topic*, first, the findings of this systematic review revealed that there was an impetus for further studies that have to be conducted, be it just descriptive studies at the beginning, and later developed into more experimental studies that could verify where we are now regarding the ethical threat there are when using AI in education. Second, it clearly shows that the AI-related issues in mobile apps for education are still a big unknown but it somehow suggests, when working with recent sources, what could the possible theoretical framing and practical consequences be of the AI-driven environment. And finally, it also stresses that the only possible perspective on the topic must always be multidisciplinary. The reason for this approach is that it can never be only evaluated by the information specialist, a designer, a teacher, or a user if the implementation of AI is relevant, dangerous, or beneficial, but it must always be a consensual evaluation of the status quo, and from this point, it is necessary to proceed further to ensure safety and security related to data, individuals and the whole society.

Thus, the contribution of this study consists in emphasizing the need for joint cooperation and research of all stakeholders when using AI-driven mobile technologies in education with special attention to the ethical issues since the present research based on the review studies is scarce and neglected in this respect.

## Author contributions

All authors listed have made a substantial, direct, and intellectual contribution to the work and approved it for publication.
